# A redox-sensitive JNK-CHOP signaling axis drives running stress-induced mtUPR activation in aged skeletal muscle

**DOI:** 10.1093/geroni/igaf122.4054

**Published:** 2025-12-31

**Authors:** Grant Laskin, Baylah Mazonson, Yuhoung Kim, Laura Verdi, Ladora Thompson

**Affiliations:** Boston University, Boston, Massachusetts, United States; Boston University, Boston, Massachusetts, United States; Boston University, Boston, Massachusetts, United States; Boston University, Boston, Massachusetts, United States; Boston University, Boston, Massachusetts, United States

## Abstract

Mitochondrial dysfunction and impaired proteostasis are aging hallmarks. The mitochondrial unfolded protein response (mtUPR) is a transcriptional program that restores mitochondrial integrity following proteotoxic stress, yet its regulation during physiological stress in aged muscle remains poorly defined. Here, we combine in vivo and in vitro approaches to investigate mtUPR regulation in aged skeletal muscle. Young (4-mo) and older (22-24-mo) C57BL/6 mice of both sexes either remained sedentary or performed three consecutive sessions of treadmill running to exhaustion as a physiological mitochondrial stressor. Running induced the putative mtUPR chaperones mtHsp70 in skeletal muscle of older mice and Hsp60 only in older males. mtUPR activation coincided with age-emergent indices of impaired mitochondrial proteostasis, most pronounced in males. Aged muscle exhibited modest carbonylation localized at intermyofibrillar mitochondria and running stress elevated 8-OHdG levels in males, suggesting greater oxidative burden in older males. To assess the role of mitochondrial reactive oxygen species (mtROS) in mtUPR activation, we induced mitochondrial matrix protein misfolding in C2C12 myotubes with or without an mtROS scavenger or stimulator. mtROS modulation amplified (stimulator) or suppressed (scavenger) the mtUPR during proteotoxic stress, but altering mtROS alone was insufficient to adjust mtUPR activation. Pathway screening revealed redox-sensitive activation of JNK-CHOP signaling, consistent with in vivo observations. Adenoviral-mediated shRNA CHOP knockdown and pharmacological JNK inhibition confirmed this axis as an mtROS-sensitive driver of the mtUPR. These findings support a model in which proteostatic and oxidative burdens in aged muscle mitochondria, especially in males, potentiate physiological stress-induced mtUPR activation via a JNK-CHOP-dependent mechanism.

